# Post‐Dengue Deep Vein Thrombosis Complicated by Pulmonary Embolism: A Case Report

**DOI:** 10.1002/ccr3.73642

**Published:** 2026-10-08

**Authors:** Sarah Nisar, Hamza Mushtaq, Muhammad Haris Khan, Fatima Sajjad, Abdullah Afridi, Noor E Fatima, Kamil Ahmad Kamil

**Affiliations:** ^1^ Department of Medicine Khyber Teaching Hospital Peshawar Pakistan; ^2^ Department of Medicine Sebring Advent Health Florida USA; ^3^ Department of Medicine Saidu Medical College Swat Pakistan; ^4^ Department of Medicine Khyber Medical College Peshawar Pakistan; ^5^ Nasrat Bashir Curative Hospital Kandahar Afghanistan

**Keywords:** case report, deep vein thrombosis, dengue fever, pulmonary embolism, venous thromboembolism

## Abstract

Dengue fever is commonly associated with bleeding complications, particularly in patients with thrombocytopenia. However, thrombotic events, such as venous thromboembolism, are rare and under‐recognized manifestations, particularly during the recovery phase, posing diagnostic and therapeutic challenges. We report the case of a 25‐year‐old previously healthy male who presented with high‐grade fever, myalgia, and headache and was diagnosed with dengue fever based on positive NS1 antigen testing. Dengue infection was confirmed by a positive NS1 antigen test performed on Day 3 of illness, consistent with early acute dengue infection. The diagnosis was further supported by the patient's clinical presentation, including high‐grade fever, myalgia, and headache in the appropriate epidemiological context of a dengue‐endemic region. During the recovery phase of the illness, he developed painful swelling of the right lower limb and progressive dyspnea. Investigations revealed markedly elevated D‐dimer levels, right lower limb deep vein thrombosis on Doppler ultrasound, and bilateral pulmonary emboli with right ventricular strain on CT pulmonary angiography images. Although dengue‐associated thrombosis has been reported in the setting of thrombocytopenia, as described by Ghatak et al., the present case demonstrates that thrombotic complications may also occur despite preserved platelet counts, highlighting the potential contribution of endothelial dysfunction and inflammatory activation. This case highlights the paradoxical coexistence of thrombosis and bleeding risk in dengue fever. To avoid potentially fatal thromboembolic complications in patients with dengue, early diagnosis, prompt anticoagulation, and a high index of suspicion are crucial.

## Key Clinical Message

Venous thromboembolism is an uncommon but important complication of the recovery phase of dengue infection. Clinicians should maintain a high index of suspicion for thrombotic events in patients with dengue presenting with new limb swelling or respiratory symptoms, even with preserved platelet counts and in the absence of classical bleeding manifestations.

## 1 Introduction

Dengue fever is a mosquito‐borne viral infection and a major public health concern in tropical and subtropical regions, with an estimated 390 million infections worldwide annually [1]. The clinical manifestations of dengue encompass a spectrum from asymptomatic infection and mild undifferentiated febrile illness to life‐threatening complications such as dengue hemorrhagic fever and dengue shock syndrome, characterized by plasma leakage, severe bleeding, and multi‐organ dysfunction [2, 3]. Although bleeding manifestations have been a clinical focus in dengue infection, the pathogenesis of dengue fever with regard to hemostatic equilibrium shifts is far less understood [4]. Although there have been increasing case reports of dengue in the literature, the presence of venous thromboembolism (VTE), including deep vein thrombosis and pulmonary embolism, is rarely reported and remains a poorly recognized complication [4].

Because dengue is primarily associated with bleeding complications and thrombocytopenia, VTE may be overlooked when patients develop vague symptoms during the recovery phase [1, 4]. The clinical complexities of dengue infections are magnified by the dual presence of thrombotic and hemorrhagic tendencies, especially when it comes to the dilemmas of anticoagulation in the context of concomitant thrombocytopenia [3, 4].

We report the case of a 25‐year‐old man diagnosed with dengue fever who developed bilateral pulmonary emboli during the recovery phase due to deep vein thrombosis in the right lower limb. This case illustrates the need for diagnostic vigilance in this serious but preventable phenomenon.

## 2 Case History

A 25‐year‐old Asian man presented to the emergency department with a history of high‐grade fever for 3 days, accompanied by headache, retro‐orbital pain, and generalized body aches. The fever had an abrupt onset, remained continuous, and was documented at up to 103°F. He did not report vomiting, abdominal pain, diarrhea, cough, hemoptysis, or any bleeding manifestations. There was no previous history of chronic medical illness or prior surgery, and his family history was unremarkable. He denied smoking, alcohol consumption, or recreational drug use.

On examination, the patient appeared ill and febrile. His pulse rate was 110 beats/min, and his blood pressure was 115/80 mmHg. No peripheral signs suggestive of chronic systemic disease were observed. Cardiovascular examination revealed normal heart sounds without murmurs; respiratory examination showed normal vesicular breath sounds with clear lung fields bilaterally, abdominal examination was unremarkable, and neurological assessment showed that the patient was alert, oriented, and without signs of meningeal irritation.

## 3 Investigations and Laboratory Findings

Initial laboratory evaluation revealed a hemoglobin level of 13 g/dL. The total leukocyte count was elevated at 12,000/μL upon admission, which subsequently declined to 4600/μL. Although leukopenia is more commonly observed in dengue infection, transient leukocytosis may occur in the early phase of the illness or in the presence of secondary bacterial infection, approaching the normal range during the course of hospitalization. The platelet count at the presentation was 192,000/μL. Peripheral blood smear examination revealed normocytic, normochromic red blood cells, with no abnormal cells noted. Microbiological testing confirmed dengue infection, with a positive NS1 antigen, while testing for malaria parasites was negative. Viral serology using ELISA was negative for other viral infections.

The coagulation profile was within normal limits; however, D‐dimer levels were markedly elevated, measuring 9968 ng/mL initially, with a subsequent decline to 4068 ng/mL, indicating an ongoing thrombotic process. Serum electrolytes were within normal limits, and renal function was preserved, with a serum creatinine level of 0.7 mg/dL. Liver function tests remained within the normal range throughout the admission period. Inflammatory markers were significantly elevated, with a procalcitonin level of 0.57 ng/mL (normal: < 0.1 ng/mL), C‐reactive protein of 142 mg/L (normal: < 10 mg/L), and an erythrocyte sedimentation rate of 54 mm/h (Normal: 15–20 mm/h), indicating a heightened inflammatory state.

The patient initially presented with fever for 3 days. On the sixth day of hospitalization (approximately the ninth day of illness), he developed sudden painful swelling of the right lower limb, accompanied by progressive shortness of breath. The swelling extended from the calf to the thigh and was associated with difficulty in movement of the leg. He also complained of shortness of breath on exertion, which was not associated with orthopnea or paroxysmal nocturnal dyspnea. Local examination of the right lower limb revealed visible swelling extending up to the groin, mild redness, warmth, tenderness upon palpation, and an increase in calf and thigh circumference. The peripheral pulses were intact.

At this time, the patient remained hemodynamically stable, except for mild tachycardia. His blood pressure was 105/70 mmHg, oxygen saturation was 93% on room air, and temperature was 99°F. In view of the acute unilateral limb swelling and respiratory symptoms, further evaluation was performed. Laboratory investigations revealed elevated D‐dimer levels, and electrocardiography demonstrated sinus tachycardia. Doppler ultrasonography of the right lower limb confirmed the presence of deep vein thrombosis. Given the worsening dyspnea, a CT pulmonary angiogram was performed, which revealed bilateral pulmonary emboli and dilatation of the right cardiac chambers, indicating right heart strain (Figure 1). Elevated D‐dimer levels and sinus tachycardia on ECG also indicated the diagnosis of pulmonary embolism.

**FIGURE 1:**
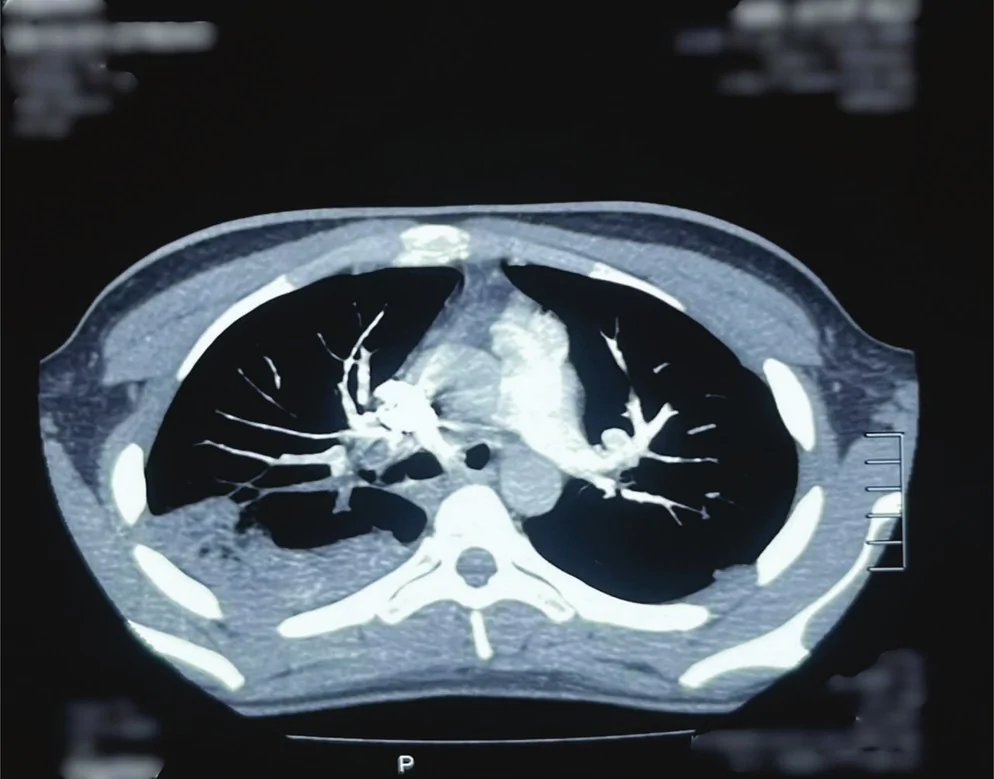
CT pulmonary angiogram showing bilateral pulmonary embolism with dilated right heart chamber.

Further investigations were performed to assess the infective and embolic sources. Chest radiography revealed a cavitary lesion in the right lung with mild cardiomegaly (Figure 2). High‐resolution computed tomography of the chest revealed a cavitary lesion in the right lung with surrounding pneumonic consolidation, minimal bilateral pleural effusion, and basal atelectatic changes (Figure 3). Additional investigations were performed to exclude alternative infectious etiologies of the symptoms. Sputum examination, acid‐fast bacilli (AFB) smear, and GeneXpert testing were negative for *Mycobacterium tuberculosis* . Blood cultures and microbiological evaluations did not identify any bacterial or fungal pathogens. These findings reduced the likelihood of other infectious causes for the pulmonary manifestations.

**FIGURE 2:**
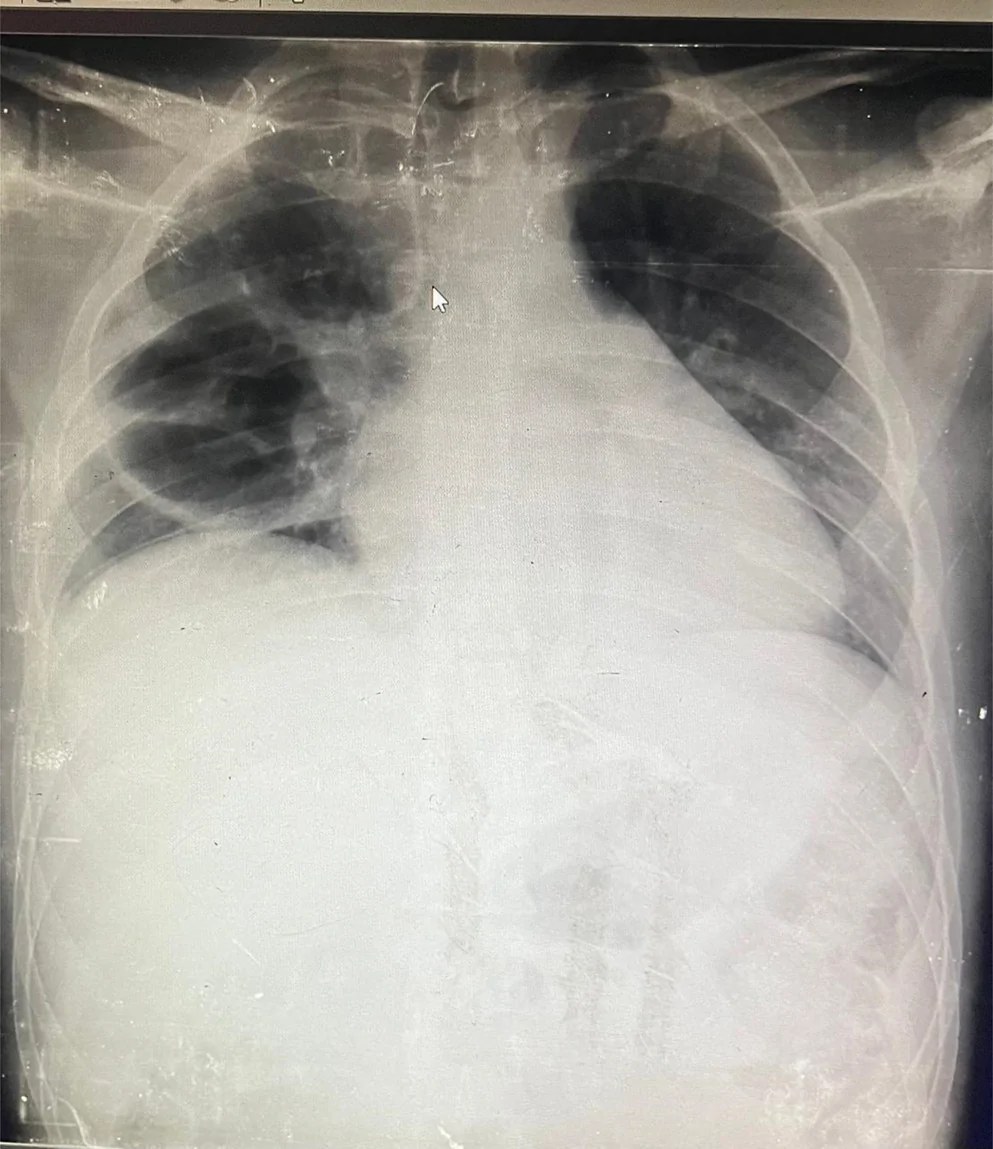
Chest X‐ray showing right lung cavitary lesion with mild cardiomegaly.

**FIGURE 3:**
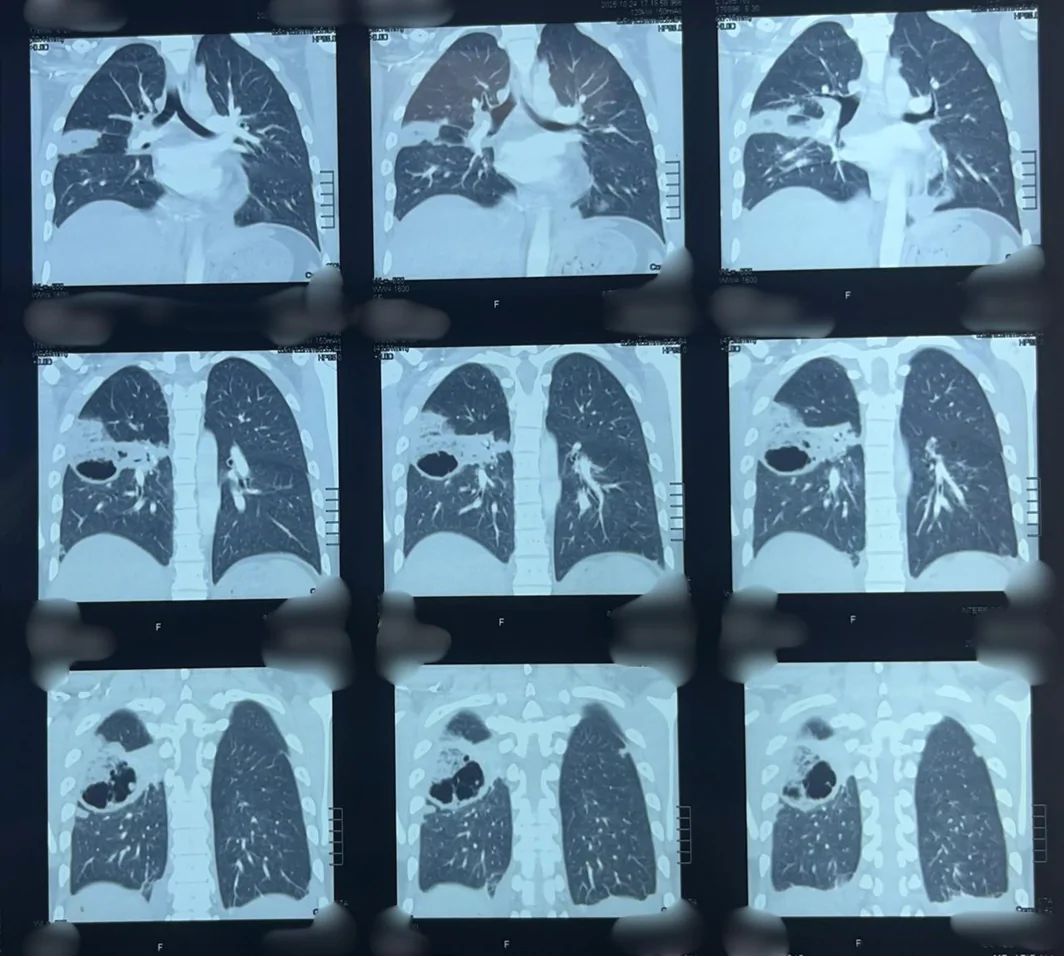
HRCT showing cavitary right lung lesion with surrounding pneumonic consolidation, bilateral minimal pleural effusion with basal atelectatic changes.

Transthoracic echocardiography revealed normal left ventricular systolic and diastolic function with an ejection fraction of 64%, no intracardiac vegetations, and a positive McConnell sign suggestive of right ventricular dysfunction. Bronchoscopy was performed to further evaluate the cavitary lesion and revealed mucopurulent secretions in the right upper lobe bronchus without an endobronchial lesion. Bronchoalveolar lavage showed no organisms on Gram stain, with bacterial and fungal cultures demonstrating no growth. AFB smear and GeneXpert testing for *Mycobacterium tuberculosis* were negative. Cytology showed no malignant cells. The available microbiological investigations did not identify a definitive infectious etiology. Given the patient's clinical improvement, no further invasive investigation of the lesion was pursued during the hospitalization.

## 4 Differential Diagnosis

Based on the clinical presentation and investigation findings, the differential diagnosis included dengue‐associated venous thromboembolism, septic pulmonary emboli, pulmonary infarction with cavitation, secondary bacterial pneumonia, and an underlying pulmonary or systemic malignancy. Given the patient's young age, absence of reported malignancy risk factors, and close temporal relationship between dengue infection and the thrombotic event, occult malignancy was considered unlikely.

## 5 Outcomes and Follow Up

The patient received intravenous paracetamol for fever control and intravenous normal saline for hydration. The patient weighed approximately 60 kg, and therapeutic anticoagulation was initiated with subcutaneous enoxaparin 60 mg twice daily, corresponding to the recommended weight‐based dosing of 1 mg/kg every 12 h. Broad‐spectrum antimicrobial therapy included intravenous meropenem (1 g three times daily) and intravenous linezolid (600 mg twice daily). For symptomatic relief, oral serratiopeptidase was administered thrice daily. Respiratory support included ipratropium bromide nebulization four times daily and beclomethasone nebulization twice daily.

The patient showed gradual clinical improvement following treatment. On the thirteenth day of admission, the patient was clinically stable and was discharged on apixaban 10 mg orally twice daily for 7 days, followed by 5 mg orally twice daily, with therapeutic anticoagulation continued for a total of 6 months. He was also discharged on oral antibiotics and cough expectorants and was counseled regarding medication adherence, gradual resumption of physical activity, prevention of recurrent thromboembolic events, and the importance of follow‐up. At outpatient follow‐up 4 weeks after discharge, the patient was clinically well, with resolution of dyspnea and right lower‐limb swelling and no recurrent thromboembolic or bleeding symptoms. Repeat Doppler ultrasonography demonstrated interval improvement of the right lower‐limb DVT, while follow‐up chest CT showed partial resolution of the cavitary pulmonary lesion. No recurrent DVT, pulmonary embolism, or subsequent malignancy was identified during follow‐up. The thrombophilia workup was also negative.

## 6 Discussion

Dengue fever is known to cause spontaneous venous thrombosis, which is rare but becoming more common. In the present case, thrombosis occurred during the recovery phase of dengue infection when the platelet counts were within the normal range, suggesting that endothelial dysfunction and inflammatory activation may contribute to a transient hypercoagulable state. Cavitary lung lesions are commonly associated with conditions such as tuberculosis, lung abscesses, fungal infections, and malignancies, all of which may independently predispose patients to thrombosis. However, in the present case, extensive investigations, including AFB smears, GeneXpert testing, and microbiological cultures, did not support these diagnoses. Furthermore, the temporal relationship between dengue infection and the onset of thrombotic complications suggests that a possible dengue‐associated prothrombotic state is the primary underlying mechanism. Cavitary lung lesions observed on imaging may represent pulmonary infarction secondary to pulmonary embolism. Although cavitation following pulmonary infarction is uncommon, it has been reported in cases of significant vascular obstruction. Bronchoscopy revealed mucopurulent secretions, raising concern for infection; however, BAL microbiology and cytology were negative. Thus, the cavitary lesion remained of uncertain etiology, with pulmonary infarction, septic embolism, and secondary bacterial infection considered.

Given the patient's young age and apparently unprovoked VTE, thrombophilia was considered. However, the thrombophilia workup was negative, supporting the possibility of a transient infection‐associated prothrombotic state. Occult malignancy should also be considered in unexplained VTE. Janoud et al. reported DVT as the presenting feature of uterine leiomyosarcoma, highlighting this association [5]. In our patient, his young age and close temporal relationship with dengue made occult malignancy less likely, although it remains an important consideration in unexplained or recurrent VTE.

Approximately 9 days after the onset of fever, the patient developed deep vein thrombosis and bilateral pulmonary embolism. An analysis of 19 reported cases of spontaneous venous thrombosis in dengue demonstrated a median thrombosis onset of 6 days post fever onset, with serious thrombotic events generally occurring between Days 5 and 10 of the illness [5]. The temporal sequence shows that thrombotic complications often arise during the transition from the acute to the recovery phase of dengue infection.

DVT in the lower limbs was the predominant thrombotic complication in this case, which is consistent with the literature. Of the 19 documented cases, 68% described lower limb DVT, most of which involved the right side with the external iliac, common femoral, and popliteal veins [5]. DVT of the right lower limb in this patient, with extension from the calf to the thigh and bilateral pulmonary embolism on CT angiography, constitutes an extreme case.

The patient's elevated D‐dimer level indicates dysregulation of coagulation cascades via several pathways: the formation of cross‐reactive autoantibodies due to molecular mimicry between viral antigens and coagulation factors, dengue NS1 protein, and resultant endothelial dysfunction, and infection‐induced tissue factor upregulation in monocytes and endothelial cells [4, 6, 7]. The associated cytokine storm augments the levels of pro‐inflammatory factors, which further worsens endothelial dysfunction and coagulation activation via tissue factor [8].

Although dengue‐associated thrombosis has been reported in the setting of thrombocytopenia, the present case demonstrates that thrombotic complications may also occur despite preserved platelet counts. Ghatak et al. described thrombosis occurring with a median platelet count of 52,000/mm^3^, exemplifying that clinically relevant venous thrombosis can occur with substantial reductions in platelets [5]. Notably, the patient did not demonstrate thrombocytopenia during hospitalization, which further emphasizes that thrombotic complications of dengue may occur even in the absence of low platelet counts.

The C‐reactive protein level indicates a more intense inflammatory response. In a prospective study, the mean CRP level was 50.53 mg/L for severe dengue and 21.62 mg/L for non‐severe cases [9]. The cavitary lung lesion was concerning for a secondary bacterial infection, as dengue increases vulnerability due to damage to the epithelium and immunosuppression [10].

The patient was initially treated with therapeutic enoxaparin 60 mg subcutaneously twice daily and was subsequently transitioned to apixaban prior to discharge, with continuation of therapeutic anticoagulation for 6 months. Among 19 spontaneous venous thrombosis cases reviewed, 57.8% received LMWH (enoxaparin); anticoagulation was well tolerated even with thrombocytopenia, and all patients had an uneventful recovery [5]. Nevertheless, the current literature describes the absence of guidelines outlining definitive courses of action for the management of dengue‐associated PE. This, coupled with the high risk of bleeding, makes the decision to use anticoagulation particularly difficult. Empirical broad‐spectrum antibiotics were initiated because of the presence of a cavitary lung lesion and concern for possible secondary bacterial infection, despite negative initial microbiological investigations.

Systematic assessment through duplex ultrasonography, CT angiography, echocardiography, and bronchoscopy was needed to tackle the expansive differential diagnosis of DVT with PE, septic emboli pneumonia, and cellulitis with sepsis. Bronchoscopy was not pursued further as the patient showed clinical improvement with ongoing management, and the potential procedural risks, including bleeding in the context of dengue infection, were considered to outweigh the expected diagnostic benefit. Given the documented bilateral PE and RV dysfunction, the patient is at risk of chronic thromboembolic pulmonary hypertension, which necessitates an extended duration of anticoagulation therapy and additional monitoring.

## 7 Conclusion

This case highlights venous thromboembolism as an uncommon but clinically significant complication that may occur during the recovery phase of dengue infection. Clinicians should remain vigilant for thrombotic symptoms in patients with dengue, as early recognition and prompt anticoagulation can prevent potentially life‐threatening outcomes. While the clinical situation is quite complex owing to the absence of standardized guidelines, the main elements of recognition, timely diagnosis, and early anticoagulation are crucial.

## Author Contributions

**Sarah Nisar:** conceptualization, supervision, writing – original draft. **Hamza Mushtaq:** conceptualization, writing – review and editing. **Muhammad Haris Khan:** conceptualization, investigation. **Fatima Sajjad:** conceptualization, writing – original draft. **Abdullah Afridi:** conceptualization, investigation. **Noor E Fatima:** conceptualization, project administration, visualization. **Kamil Ahmad Kamil:** conceptualization.

## Funding

The authors have nothing to report.

## Consent

The authors certify that they have obtained all appropriate written patient consent forms. In the form, the patient has given his consent for his images and other clinical information to be reported in the journal. The patient understands that his name and initials will not be published, and due efforts will be made to conceal his identity.

## Conflicts of Interest

The authors declare no conflicts of interest.

## Data Availability

The data that support the findings of this study are available on request from the corresponding author. The data are not publicly available due to privacy or ethical restrictions.
